# Percutaneous Fine Needle Biopsy in Pancreatic Tumors: A Study of 42 Cases

**DOI:** 10.1155/2012/908963

**Published:** 2012-12-12

**Authors:** Piotr Lewitowicz, Jaroslaw Matykiewicz, Jacek Heciak, Dorota Koziel, Stanisław Gluszek

**Affiliations:** ^1^Department of Pathology, University of Jan Kochanowski (UJK), Al. IX Wieków Kielc 19, 25-317 Kielce, Poland; ^2^Department of Surgery and Surgical Nursing, University of Jan Kochanowski (UJK), Al. IX Wieków Kielc 19, 25-317 Kielce, Poland; ^3^Department of Radiology, Joint Provincial Hospital Kielce, ul. Grunwaldzka 45, 25-736 Kielce, Poland

## Abstract

The technological progress within the range of methods of pancreas imaging and their more common accessibility selects a group of patients requiring a microscopic diagnosis. Percutaneous fine needle aspiration biopsy under the control of ultrasonography (PCFNA/USG) is the method commonly used in determining the character of a focal pancreatic lesion. *Aim of the Work*. An assessment of the accessibility of PCFNA biopsy in the assessment of solid and cystic changes in a pancreas and the correlation of the results of imaging examination, cytological smear and concentration of a serous marker CA19-9. *Material and Methodology*. In our material we analysed 43 cases of tumors of the pancreas among the patients who were at the average age of 59 ± 10.4 (14 women, 28 men) diagnosed by PCFNA biopsy. *Results*. In a group we are 23 cases of cancer, 12 cases of inflammation and 7 cases of cellular atypia for which 2 cases of IPMN were included. The sensitivity of the method was 92.5% but specificity was 68%. In our opinion PCFNA/USG is a method of the comparable sensitivity and specificity with fine needle aspiration biopsy with EUS control and its efficiency depends to a considerable degree on experience and interdisciplinary collaboration.

## 1. Introduction

Tumors of the pancreas are a difficult problem of contemporary medicine. They are very often diagnosed in the advanced stage because after they reach the right size, they cause clinical symptoms, such as, stomachache, backache, yellowish discoloration of integument or nausea, and vomits. At the time of growing accessibility to modern radiological imaging techniques often and often are found non-characteristic, often small focal lesions which require the further diagnostic. This allows selecting a group of patients with more often recognized precancerous changes or early stages of pancreatic cancer without clinical symptoms of the disease. The first examination which is done most often through percutaneous ultrasound examination of the abdominal cavity (USG), in which the accuracy in defining the character of focal lesions of the pancreas is 50–70%. The accuracy of this method is improved by applying endoscopic USG (EUS), the Doppler method, USG 3D, and intraoperational USG. During the ultrasound examination both percutaneous and endoscopic we can additionally perform fine needle aspiration biopsy (FNA/USG) of a tumor of the pancreas receiving material for cytological examination [1, 2].

According to the character in imaging examinations, focal lesions of the pancreas is divided into solid and cystic tumor. In both groups there are inflammation processes, benign neoplasms, and malignant neoplasms. Because of difficulties in defining the kind of lesions we deal with, only the correlation of clinical symptoms, the result of the imaging examination, cytological or/and histopathological, and the concentration of neoplastic markers manage with some possibility to indicate the type of the lesions. It influences the way of the further therapeutic management.

For the defined group of patients with the clinically unnoticeable as well as the evident tumorous lesions, using this technique in order to get material for a cytological examination is quite a simple solution. PCFNA biopsy is a method which is safe, cheap, and demonstrates big sensitivity and specificity concerning both solid and cystic tumors under the condition that it is performed in an interdisciplinary way [3].

The aim of the study was to assess accessibility of PCFNA biopsy in the assessment of solid and cystic changes in the pancreas and the correlation of the results of imaging experiments, cytological smear, and concentration of a serous marker CA19-9.

## 2. Material and Methodology

The research was conducted in a forward-looking way between 2009 and 2011. The analysed a group consisted of 43 patients with focal lesions in the pancreas diagnosed in the Clinical Ward of General, Oncological and Endocrinological Surgery of the Joint Provincial Hospital in Kielce. Patients with the focal lesions of the pancreas recognized in the USG and CT (computed tomography) examination of the abdominal cavity were qualified for the research.

Percutaneous ultrasonography of the abdomen was performed based on the clinical symptoms. Jaundice, gastric obstruction, abdominal or back pain, and weight loss were the main symptoms which were reported by patients. Most patients suffered from fatigue. Jaundice, nausea, and vomiting were common among patients with a tumor localized in the head of pancreas (33 pts), while abdominal or back pain and weakness were present in patients with a tumor of the body of pancreas (9 pts). Some patients had a palpable mass in the abdominal cavity. USG was performed as first diagnostic imaging.

Patients with typical clinical symptoms and imaging changes of inflammation and postinflammation processes of the pancreas were excluded from the analysis. Before performing PCFNA/USG, the patients had percutaneous ultrasonographic examinations as well as CT of the abdominal cavity with a contrast agent performed. Most of the patients had the determined concentration of the CA19-9 antigen in blood serum. The decision of performing PCFNA biopsy/USG was undertaken based on the results of the imaging examinations, qualifying for a biopsy the tumors: solid, cystic with tissue echoes, cystic with dividing walls and calcification. Before performing FNA biopsy the patient's consent to examination was received.

The team which consisted of a radiologist, a pathologist, and a surgeon performed a puncture under the control of USG model ESATOE My Lab Classic C making use of the dynamic character of an examination, the possibility of visualization of blood vessels in the vicinity of the tumor and the possibility of correction of the route of a needle and defining the localization of the end of a needle in the lesion. The needles 22G or 25G of the length of 9 cm were used depending on the conditions of reaching the tumor (the depth of the lesion localization, its size, and echogenicity, the thickness of abdominal integument and visceral lipomatosis). The part of the lesions, mainly cystic ones, underwent another puncture depending on the kind of a tumor and aspirated material. Routinely solid tumors were diagnosed with a needle 22G but cystic tumors were aspirated twice with a needle 25G (low risk of post biopsy fistulas) because according of the kind of liquid the additional material was taken from the intra-cystic tissue echos. Smears on the basic small pieces of glass were fixed in 96% of ethanol and then dyed with hematoxylin and eosin, in some cases with mucicarmine to check the presence of acid *mucopolysaccharides* or cell blocks and immunohistochemistry.

The cytological lesions were grouped in three categories:

1. *Cancer*: the evident cytological characteristics of a cancer.
2. *Atypia and inflammation*: among the exponents of inflammation there are cellular populations with atypia in a degree from the small one to the big one without unequivocal characteristics of a cancer.
3. *Inflammation*: inflammation without the presence of the atypical cells.

The results of the imaging examinations, the concentration of neoplastic markers, and the cytological research were compared to each other in order to define the relationship between them. The statistical analysis was carried out based on the STATISTICA software with the Anova Kruskal-Wallis's test and Spearman's rank correlation.

## 3. The Result of the Study

**Figure 1:**
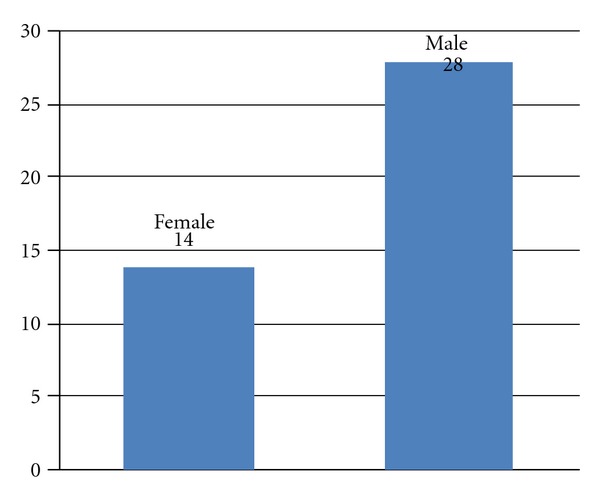
Sex of the patients.

**Figure 2:**
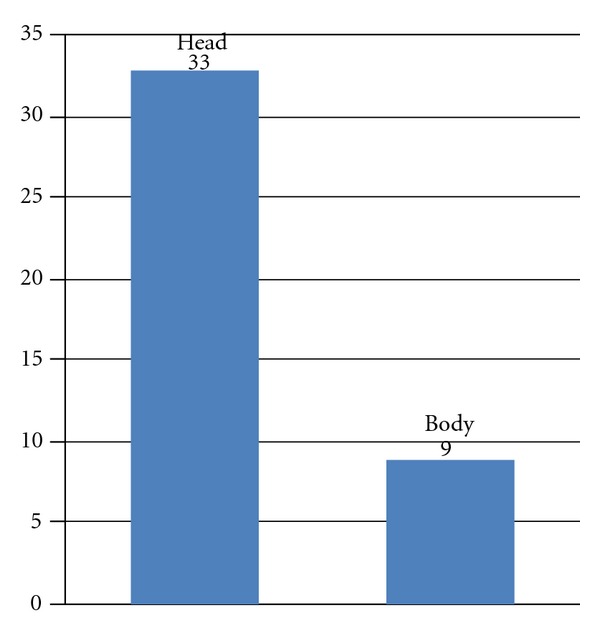
Localisation of the tumor.

In the examined and analysed group there were 14 (33.3%) women and 28 (66.6%) men at the age of 36 to 82 (Figure 1). The average age of the patients was 59 years. The tumors were placed in the head of the pancreas (33 patients—78.5%) and the body of the pancreas (9 patients—21.5%) (Figure 2). The average size of the focal lesions was 2.5 ± 0.9 cm. The diagnostics material during PCFNA biopsy was taken in 42 cases. In one case because of the considerable obesity and visceral adipositas there was no visualization of a needle in the lesion and the examination was regarded as nondiagnostic.

12 inflammations, 7 lesions with atypia and inflammation, and 23 cancers were diagnosed on the basis of FNA/USG biopsy examinations.  Table 1 shows the characteristics of the patients with taking into consideration the age and sex of the patients and the type of tumorous lesion.

**Figure 3:**
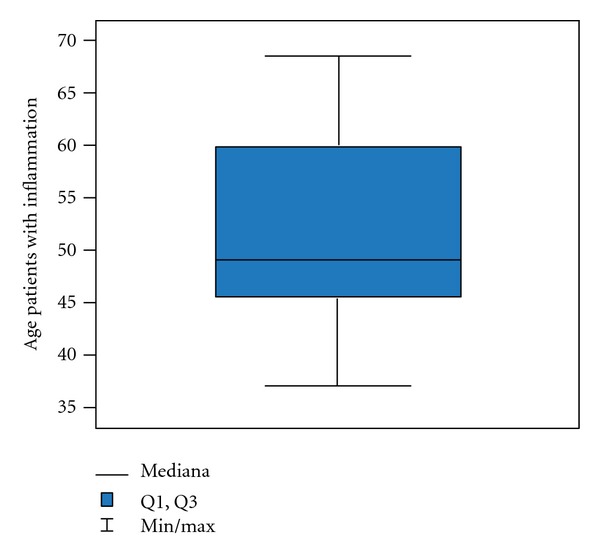
Age in group with inflammation.

**Figure 4:**
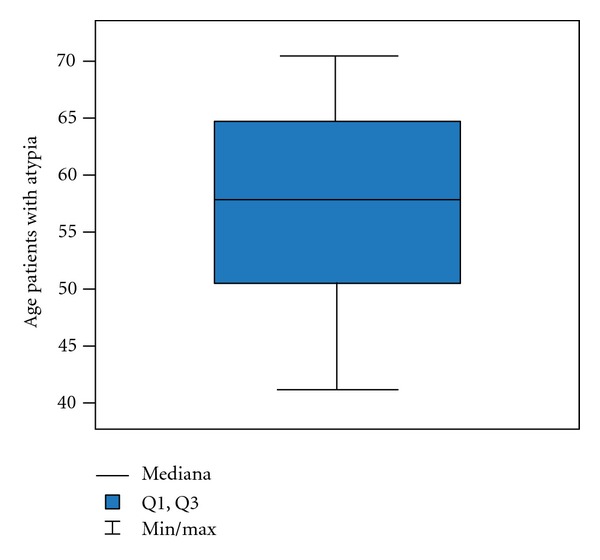
Age in group with atypia.

**Figure 5:**
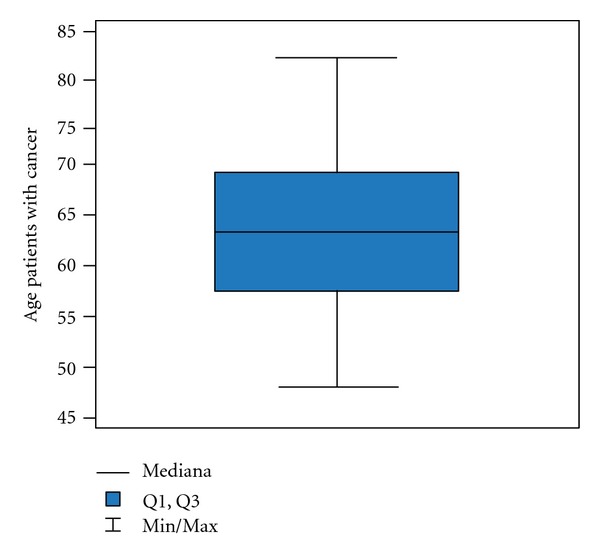
Age in group with cancer.

Generally the average age of the patients in the examined group is 59 ± 10.4 years old but with a group with inflammation 51 ± 9.8 years old (Figure 3); in a group with atypia and inflammation 57 ± 10.6 years old (Figure 4) and in a group with a cancer 64 ± 8.5 years old (Figure 5).

**Figure 6:**
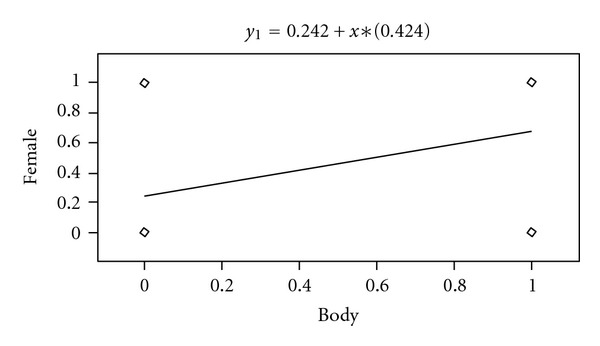
Linear relationship body localisation of tumor with female.

The analysis of Spearman's correlation of the sex with the tumor localization shows the considerable dependence of female with the localization of the tumor in the body of the pancreas *P* = 0.0160 (Figure 6). The similar dependence was not observed for tumors of the head of the pancreas depending on the sex.

**Figure 7:**
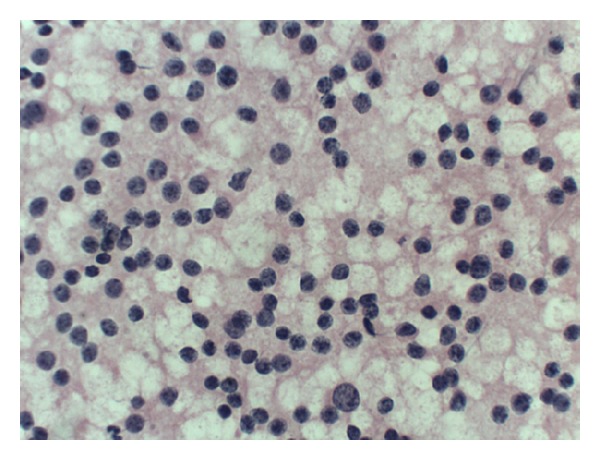
Neuroendocrine carcinoma (typical “salt and peper” chromatin). H-E 40X.

**Figure 8:**
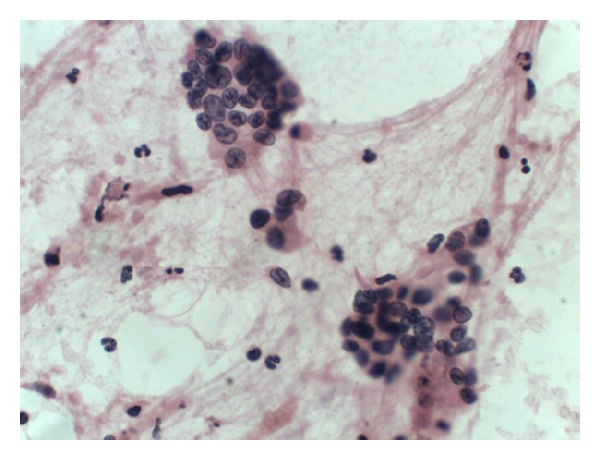
Well-differentiated ductal carcinoma H-E 20X.

**Figure 9:**
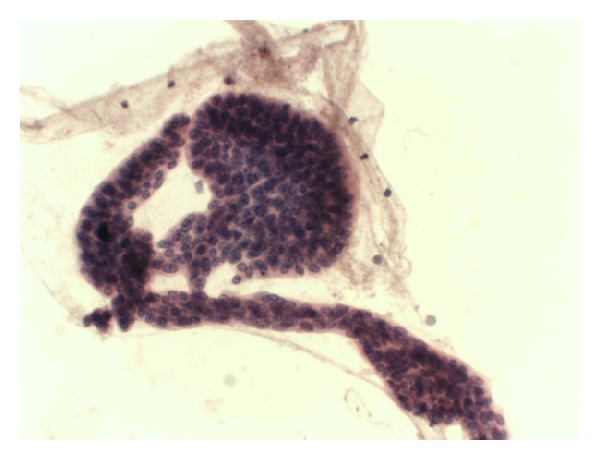
IPMN with low-grade dysplasia. Regular sheet columnar epithelium and mucoid background H-E 20X.

**Figure 10:**
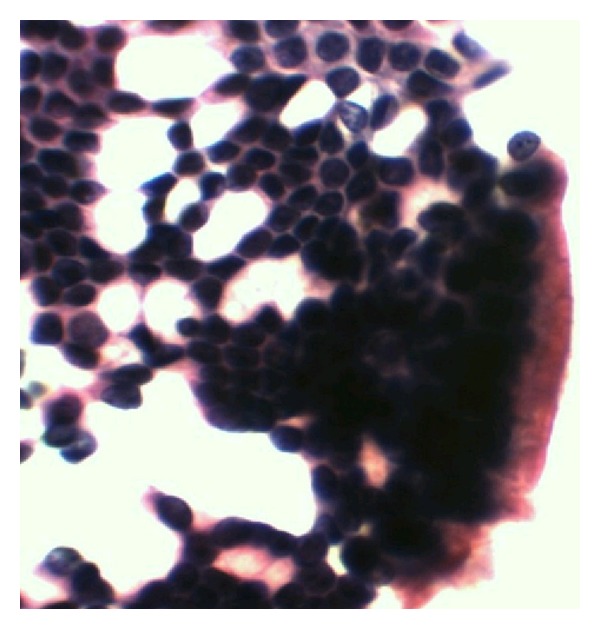
IPMN with high-grade dysplasia. Cribriforme epithelial hyperplasia. Mucikarmin 40X.

In the 23 cases typical cytological characteristics of the cancer were diagnosed (20 cases of ductal caricinoma, 1 of mucinous carcinoma, and 2 neuroendocrine carcinoma) (Figures 7 and 8). In 12 cases the cytological characteristics of inflammation without the presence of epithelial cells with atypia, but in 7 cases they determined the characteristics of inflammation and the presence of atypical epithelial cells (Table 2). In this group, out of 7 cases classified as the atypical lesions there are two cases of IPMN with a dysplasia of a considerable degree without certain cytological exponents of the coexisting cancer (Figures 9 and 10).

Taking into consideration the data from PCFNA biopsy and clinical data, the cancer of the pancreas was finally recognized in 25 patients. In 23 based on PCFNA biopsy and in 2 based on the clinical course of a disease. In 2 people for whom a cancer was diagnosed based on metastasis changes in the liver discovered in the imaging examinations based on PCFNA biopsy, inflammatory lesions with atypia were stated. In this group there are both cases of IPMN.

Among 23 patients with a cancer diagnosed on the basis of PCFNA biopsy/USG examinations there were not any removals of a section of an organ.

All the cancers were classified according to cTNM (clinical TNM) classification. Preoperative imaging, upper gastrointestinal endoscopy, and serum concentration of CA 19-9 provided necessary information of clinical advancement of the disease. In some cases, additional information was provided by endoscopic retrograde cholangiopancreatography (ERCP).

The lack of possibility of radical treatment was stated on the basis of the results of the imaging examinations in 11 patients (the characteristic T3/T4 and M1), ERCP in 2 patients, and laparotomy in 9 patients. The characteristic which disqualified the patients from treatment with the use of resection were metastases in the liver or peritoneum, infiltration of the tumor on a superior mesenteric vessels, or a portal vein. The removal of a section of an organ was performed in one case when inflammation with atypia was recognized where based on PCFNA biopsy/USG and in a postsurgical examination weaving of adenocarcinoma was stated.

Palliative procedures, a double bypass was performed when a laparotomy was necessary to evaluate unresectability of the tumor. Biliointestinal anastomoses in the 3 cases and gastrointestinal anastomoses in the 9 cases were performed when it was possible. In the 2 cases, endoscopic biliary drainage was performed.

The assessment of the analysis of sensitivity and specificity of the results depends closely on the classifications of cytological lesions depicted as “with atypia”. Assuming that cytological atypia is treated as a positive examination, the sensitivity was 75% and specificity was 29%. Because of the fact that cytological atypia is not connected with determining recognition and then purposeful treatment, in the research we acknowledged changes with cytological atypia as the negative results although we were aware of two cases of IPMN and one case of a cancer. For such a formulated rule the sensitivity was 92.5% but the specificity 68%.

The analysis of the result of the cytological research with a USG/CT image shows a close correlation at the level *P* < 0.0001 of unequivocal recognition of a cancer with a solid tumor, hypoechogenic in the USG examination. The cystic tumor with tissue echoes in the USG examination shows a considerable correlation with cytological lesions depicted as unspecified atypia, including 2 cases of IPMN *P* = 0.00017. 

The inflammatory lesions appeared only in the head of the pancreas with the coincident frequency among women and men and in the correlation of the USG image (cystic tumors without solid mural parts, periductal hypoechogenic signs)showed the similar correlation for each sex (*P* = 0.035).

In the group with a cancer the average value of the concentration CA 19-9 was 1082 ± 1526 U/mL; in the group with atypia was 973 ± 1631 U/mL, but in the group of inflammation was 20.6 ± 26 U/mL. There was not a considerable difference of the value of CA19-9 when the groups with a cancer and atypia were compared but when they were compared with the group with inflammation there was a considerable difference (*P* < 0.0001).

## 4. Discussion

Among the majority of the patients qualified for pancreas cancer resection evaluated cytological or histopathological procedures with different sensitivity, specificity, and potential complications are conducted. The lack of microscopic sure verification kind of a tumor in the 4–6-week observation and characteristic clinical image as well as lesions in the imaging examinations (USG, CT, NMR, PET) are sufficient factors which prove the need of the resection of the pancreas.

PCFNA biopsy was mostly performed in those patients for whom there was a high possibility of not performing resection of a tumor. This is a method with sensitivity and specificity over 90% for a cancer of the solid type, however specificity lowers considerably for cystic lesions. A core biopsy increases considerably sensitivity and specificity of recognizing solid lesions or parts of cystic ones, however the risk of the complications of percutaneous core biopsy for the parts of lesions in the head and the body of the pancreas is significantly bigger than for FNA and is about 5%. Also the frequency of the complications, namely, postbiopsy pancreatitis, bleeding, pathologic fistulas and infection is considerably bigger during FNA cystic lesions and FNA under the control of EUS [3–10], Laparotomy and core biopsy or the cuneiform resection of a tumor under the control of eyesight characterizes the biggest sensitivity and specificity, however it is the most invasive with a high risk of complications, expensive which requires at least several days of hospitalization.

PCFNA biopsy has a bigger specificity, and at lower frequency in comparison with taking brush swabs during the endoscopic retrograde cholangiopancreatography (ECPW). A brush biopsy is a kind of an examination which enables to identify a group of patients with lesions of dysplastic ductal epithelium like a pancreatic intraepithelial neoplasia (PaIN)s and IPMN), however differentiation between atypia/cytological dysplasia with a regeneration atypia is often very difficult [3, 11, 12].

In the case of the solid tumors of the head and the body of the pancreas, PCFNA biopsy is a method with a sensitivity and specificity of 90–100% depending on the size and localization of a tumor, a method of biopsy and experience of the team who performed this examination. The kind of tumors which causes diagnostic problems and are the causes of differences in a strategy of dealing with these problems between the environment of gastrologists and surgeons are tumors of the cystic types [13–17].

The aspirated biopsy under the control of EUS is the best tool for the cytological assessment of pancreas carcinoma especially with localization in head. Usefulness this method in tumor of the corpus and tail of pancreas is very limited. Harewood demonstrated the presence of tumorous lesions in the pancreas in 185 patients where the previous biopsies performed in the technique of guiding in CT or during ECPW gave a negative result. PCFNA biopsy/EUS had sensitivity of 94% and thoroughness of 92% for discovering pancreas carcinoma among the patients with diagnostically negative results of the examination of the material taken during ECPW and the sensitivity of 90% and 84% of thoroughness among the patients with negative biopsies under the control of CT [11]. In the examined group the sensitivity of the method was similar although the examination was carried out with the use of percutaneous USG and was 92.5%. 

We demonstrated the high sensitivity of the imaging examinations (USG and CT) in the assessment of solid hypoechogenic/hypodense solid lesions. In our material each case like this was treated with the suspicion of a cancer. The radiological changes depicted as a cystic tumor with tissue echoes demonstrated the dependence (*P* = 0.00017) with cytological atypia in smears and included two cases of IPMN. 

The criteria of the tackling cystic tumors but not the mucoid ones, which were established in 2006, select the certain group of the cystic tumors which requires the special diagnosis and possible surgical treatment. As indicated for the surgical treatment there are tumors >3 cm with the symptoms or tumors of the size 1–3 cm with high-grade stigmata (dilated main pancreatic duct >6 mm and mural nodule). The risk of the suspicion of a cancer is in a case: marked dilatation pancreatic duct >10 mm, large mural nodule, irregularity of the ductal wall, thickened septum-like structures. [18–20]. The role FNA/USG is discussed with reference to benefits and risks of the method, however but some is used in a routine way and also allows to recognize rare cancers of the pancreas [21, 22]. The aim of differentiating between the inflammatory lesions and tumorous ones includes the assessment of the concentration of CEA, CA19-9, and activity of amylase in the aspirated liquid from the cystic lesion of the pancreas [23]. This is valuable information in the process of differentiating inflammatory lesions and mucous cystic tumors (IPMN). Based on 116 cystic neoplasms Lahat and others determined the lack of the clinical symptoms in 27%, thus small symptoms such as pain and epigastric discomfort in 57% [24]. Pitman in his work pays attention to the specific difficulty in an unequivocal cytological assessment of mucous tumors where dependently on the degree of atypia/dysplasia the specificity is from 30 to 70% [18]. Diagnostic difficulties of the solid lesions with the possibility of the negatively positive results appear in the course of autoimmune pancreatitis (AIP), chronic pancreatitis, and PaIN [14, 25–27].

In 2011 Zubarick proposed to assume the control over the assessment of CA19-9 patients with the family history concerning the carcinoma of the pancreas. In case of the above normal concentration of CA19-9 the patients had FNA/EUS examinations of the indicated focal lesion. In this way neoplastic lesions were diagnosed in 5 patients (0.9% of the analyzed group in which no symptoms were recognized). The diagnosed lesions: 1 neuroendocrine tumour, 1 IPMN, 1 mucous neoplasm, and 1 PaIN. The carcinoma of the pancreas was diagnosed in 1 patient (0.2%) [28]. One can remember that mucous tumors, including IPMN are rarely characterized by significantly above normal CA19-9, and in our material we observed solid carcinoma with CA19-9 within the norm.

There is a worrying clinical course of the tumor of the pancreas. Clinical symptoms were not characteristic, were underestimated by patients and doctors, and late appearance of the tumor of the pancreas was common in the course of the disease. Consequently respectability of pancreatic tumors is very rare. In the group with identification of the carcinoma of the pancreas 13 patients were disqualified from surgical treatment because of the advanced stage of the local tumour (infiltration on blood vessels) or the presence of metastases in the liver. Resection was not carried out in any of the 9 patients who were treated with laparotomy. They limited it to the performing of the drainage procedures. We performed biliointestinal anastomoses in some patients with jaundice and gastrointestinal anastomoses in all patients who have laparotomies. This procedure is recommended as prevention of duodenal occlusion [29]. The late recognition of the pancreas carcinoma and lack of effects of treatment in the depicted group is bigger than in academic literature [6, 11]. It is also important to take into consideration that additionally 4 cases of the pancreas carcinoma were recognized during observation of patients, in a cytological examination inflammation with atypia were stated but a clinical course might be appropriate to the carcinoma of the pancreas.

## 5. Conclusions

1. PCFNA biopsy/USG is in connection with the imaging examinations, a sensitive and safe method of recognizing the advanced stage of the carcinoma of the pancreas.
2. The results of cytological inflammatory lesions with atypia should pay a special attention to those patients as the potential candidates for surgical treatment as for possibility of coexisting of the carcinoma of the pancreas.
3. In the diagnosing of the carcinoma of the pancreas also other clinical data such as the concentration of CA19-9, the ultrasonographic image of the focal lesion of the pancreas, and their localization may be helpful.

## Figures and Tables

**Table 1:** Type of tumor, age and sex of patients, and tumor localization.

	Age patients	Sex	Tumor localisation	
			Head	Body
Inflammation	51 ± 9.8	2 female 10 male M/F 5 : 1	12	0
Atypia and inflammation	57 ± 10.6	1 female 6 male M/F 6 : 1	7	0
Cancer	64 ± 8.5	11 female 12 male M/F *≈* 1	14	9 (7 female, 2 male)

**Table 2:** Cytological results.

	*n*	True positive (TP)	False negative (FN)
Cancer	23	23	0
	20 ductal carcinoma		
	1 mucinous carcinoma		
	2 neuroendocrine carcinoma		
Atypia and inflammation	7	21 (2 IPMN)	2 (1 IPMN with hight grade atypia and ductal carcinoma and 1 ductal carcinoma)
Inflammation	12	12	0
